# Predicting vasovagal syncope during head-up tilt test: three machine learning approaches

**DOI:** 10.3389/fninf.2026.1740746

**Published:** 2026-06-08

**Authors:** Matjaž Klemenc, Daniel Pellarini, Aleš Papič, Pavlin G. Poličar, Dejan Štepec, Zoran Bosnić

**Affiliations:** 1 Department of Cardiology, General Hospital of Nova Gorica, Šempeter Pri Gorici, Slovenia; 2 University of Ljubljana, Faculty of Computer and Information Science, Ljubljana, Slovenia

**Keywords:** analytical modeling, head-up tilt test, heart rate variability, machine learning, vasovagal syncope

## Abstract

**Introduction:**

Syncope prediction during head-up tilt testing (HUTT) remains challenging due to the complex interplay between autonomic and cardiovascular responses. This study investigates three computational approaches to forecast HUTT outcomes using continuous electrocardiogram (ECG) and blood pressure recordings from 105 patients with a history of syncope who underwent HUTT following a modified Italian protocol.

**Methods:**

Beat-to-beat heart rate and blood pressure signals were analyzed using: (1) gradient boosting models applied to frequency-domain features of heart rate variability (HRV); (2) an analytical modeling approach employing k-nearest neighbors (kNN) regression on transformed physiological signals; and (3) an incremental neural network model.

**Results and Discussion:**

Among these, the kNN regression approach provided the most consistent short-term forecasting of syncope probability, maintaining mean absolute errors below 0.13 for predictions up to 300 s before syncope onset. Gradient boosting models achieved promising classification performance with ROC AUC values up to 0.70, while the incremental network yielded moderate results. These findings demonstrate that data-driven analysis of early physiological changes can enable short-term forecasting of vasovagal syncope during HUTT, supporting the development of predictive tools for clinical risk assessment and personalized syncope management.

## 1 Introduction

A syncope is defined as a transient loss of consciousness (TLOC) due to cerebral hypoperfusion, characterized by a rapid onset, short duration, and spontaneous complete recovery (Brignole et al., 2018). Approximately one million patients are evaluated for syncope annually in the USA. It has been estimated that syncope evaluation accounts for 3–5% of emergency department visits and 1–6% of hospital admissions (Fenton et al., 2000). In Europe, about 1% of referrals to the emergency departments are due to syncope. Of these, approximately 40% are hospitalized (Blanc et al., 2002). Similar results were recently reported by SEED investigators (Reed et al., 2024). The incidence and prevalence of syncope events are similar in men and women (Ruwald et al., 2012), with the lifetime cumulative incidence being over 35 % (Ganzeboom et al., 2006).

Tilt testing has an acceptable sensitivity and specificity when these are calculated in patients with syncope or without a history of syncope (Flevari et al., 2009). The head up tilt test (HUTT) is considered positive if there are physiological changes such as bradycardia, cardiac pauses, and hypotension that occur alongside an exact reproduction of symptoms that the patient had previously experienced during their spontaneously occurring episodes (Parry et al., 2009).

In a recent systematic literature review, the overall positivity rate in patients with syncope was 66% for the glyceryl trinitrate (GTN) protocol and 61 % for the isoproterenol protocol; the respective positivity rate in subjects without syncope (controls) ranged from 11 to 14% (Forleo et al., 2013). HUTT is time-consuming and can be complicated by severe arrhythmias such as ventricular fibrillation (Blanc, 2013), necessitating the presence of adequate and trained staff for an uneventful procedure (Conte et al., 2009).

In recent years, several studies have investigated prediction and characterization of orthostatic responses and head-up tilt test (HUTT) outcomes using machine learning (ML) and data-driven approaches. For example, Gilmore et al. (2021) used random forest models derived from blood pressure and heart rate time-series measured during HUTT to categorize orthostatic intolerance phenotypes, reporting successful categorization of more than 95% of patients with a single condition and data-driven subgrouping of mixed-pathophysiology cases. Kim et al. (2020) developed heart-rate-based ML screening models for orthostatic hypotension using clinical and autonomic function test features, achieving classification accuracy up to 90.6% with a random forest model. He et al. (2021) demonstrated that early HUTT hemodynamic recordings combined with feature selection and ML classifiers can enable earlier prediction of HUTT outcome, reporting ROC AUC up to 0.94 while reducing required tilt duration. Finally, Solbiati et al. (2024) reported feasibility results on predicting orthostatic tolerance using ECG data acquired outside the tilt-test procedure, suggesting that pre-test ECG may contain predictive signatures relevant to orthostatic responses.

In our previous study, we developed a statistical model for early prediction of HUTT based on the analysis of heart rate variability (HRV) and baroreflex sensitivity (BRS). While there are discernible and meaningful differences between HUTT positive and HUTT negative subjects, they are not sufficient to discriminate between the two groups and predict a syncope early in the HUTT. We concluded that it was not likely that syncope predictors of practical value can be obtained from aggregate HRV spectral analysis and BRS values (Klemenc and Štrumbelj, 2015).

In this paper, we aim at addressing the prediction of HUTT outcome with three distinct approaches: (1) by **transforming the problem into the frequency space** using the Fast Fourier Transform and filtering the data only by the most important coefficients in the frequency space. Afterwards, we apply two machine learning algorithms to train the HUTT test outcome: random forests and logistic regression; (2) by modeling the **probability of a syncope analytically**. We engineer four intermediate features that are derived from the input data, and predict the values of the probability curve for an interval of up to 600 seconds ahead into the future; (3) by modeling the input data flow using machine learning **algorithms for incremental learning** and applying an adaptive neural network to sequential windows of data.

## 2 Materials and methods

One hundred and five patients with recurrent syncope (at least two episodes during the previous 6 months) were enrolled in the study. All participants were examined according to the ESC guidelines (Moya et al., 2009). None of the patients had carotid sinus hypersensitivity, diabetes, or coronary heart disease. Also, patients with non-sinus rhythm and with frequent ventricular or supraventricular ectopic beats were excluded from the study. The study complied with the Helsinki Declaration and was approved by the National Ethics Committee of Slovenia. All subjects gave their informed written consent.

### 2.1 Head-up tilt test (HUTT)

HUTT has been described in detail in our previous study (Klemenc and Štrumbelj, 2015). Briefly, we used a modified Italian four-phase protocol: first–stabilization (1st 5-min interval), second–passive phase (2nd to 7th 5-min interval; 65-degree tilt), third–provocative phase (8th to 10th 5-min interval; 65-degree tilt), and fourth–final phase (11th 5-min interval). GTN was administered at the end of the second phase. During the passive phase, the patient is tilted head-up to 65 degree for 30 minutes. In provocative phase patient receives sublingually 200 μg of GTN. 15 minutes after the application of GTN the patient is lowered into a horizontal position.

A positive response was defined by syncope (transient loss of consciousness associated with loss of postural tone) or near-syncope (pallor, nausea, light-headedness, diaphoresis, blurred vision) associated with the following hemodynamic changes: a 60% decrease in systolic blood pressure from baseline values or an absolute value of 80 mmHg (vasodepressor response) and/or a decrease in HR 30% from the baseline value or an absolute value of 40 beats/min (cardioinhibitory response) (Mallat et al., 1997).

### 2.2 Signal processing

ECG electrodes were attached to the skin, so three modified leads were obtained (CM1–modified V1, CM2–modified V2, and CM5–modified V5). The two leads having the most prominently expressed R-peaks were used for further analysis; ECG recordings were analyzed by Nevrokard HRV Analysis software (Nevrokard, Izola, Slovenia). Before the analysis, all recordings were checked by an experienced cardiologist and edited, if necessary, to exclude ectopic and artifact signals. The RR intervals measured before and after an ectopic beat were replaced by two interpolated RR intervals, which were calculated from a preceding and a succeeding sinus interval. No signal containing more than 1% of ectopic beats was analyzed.

### 2.3 Transformation and processing using FFT

For each patient, we extract two sets of time series, the first corresponding to the stabilization phase, and the other corresponding to the first ten minutes of the passive phase. Each set of time series includes measurements of blood pressure and ECG. After standardization (zero-centering and rescaling to standard deviation 1), we extract the Fourier coefficients of each time series, keeping only the 50 coefficients corresponding to the largest frequencies in the stabilization phase. Additionally, we compute the differences in Fourier coefficients between the stabilization phase and the first ten minutes of the passive phase. The resulting feature vector contains 300 features, where each blood pressure and ECG measurements contain 50 Fourier coefficients for the stabilization phase, 50 Fourier coefficients for the passive phase, and 50 differences in Fourier coefficients between the two phases.

This task is framed as a binary classification problem, predicting whether the syncope event will occur at any point throughout the administration of the test. We evaluate three classification models, including logistic regression and random forests as implemented in the scikit-learn library (Fabian, 2011), as well as gradient boosting as implemented in the XGboost library (Chen and Guestrin, 2016).

We report the receiver operating characteristic area under the curve (ROC AUC) and accuracy scores. ROC AUC was selected as the primary performance metric due to the moderately imbalanced class distribution (52 positive vs. 53 negative cases) and the clinical relevance of threshold-independent evaluation. The evaluation is performed using the leave-one-out cross-validation and computes the 95% confidence intervals (95% CI) using the DeLong method (DeLong et al., 1988) for the ROC AUC score, and Wilson's method (Wilson, 1927) for the accuracy score. Unlike accuracy, which depends on a specific decision threshold and may be unstable in small cohorts, ROC AUC provides a global measure of discrimination across all possible thresholds. We therefore treat the ROC AUC as the primary metric of interest and report the accuracy only to facilitate comparisons to other reported methods. We assess differences between methods using DeLong's test for correlated ROC curves. P-values were corrected for multiple comparisons using the Benjamini-Hochberg false discovery rate (FDR) procedure.

### 2.4 Analytical Modeling of Syncope probability

We outline the analytical framework developed to predict syncope probability by leveraging a k-nearest neighbors (K-NN) machine learning approach. Our objective is to transform patient data into a continuous signal that represents the immediate probability of a syncope event and then forecast the relative difference between a baseline measurement and its future value. The baseline is determined during the stabilization phase of the tilt test.

According to Raviele and Alboni (1994), syncope can be determined based on four distinct hemodynamic criteria, e.g., a 60% decrease in systolic blood pressure from baseline values or an absolute value of 80 mmHg (vasodepressor response) and/or a 30% decrease in heart rate from the baseline value or an absolute value of 40 beats per minute (cardioinhibitory response)–each expressed in relative form.

Equations Equation 1–Equation 4 define the transformation process, where Equations Equation 1–Equation 2 refer to blood pressure, and Equations Equation 3–Equation 4 refer to heart rate. The index equation (Equation Equation 3) refers to measurements taken during the stabilization phase, while Equation Equation 4 refers to the time of observation.


$$\begin{array}{l} p_{1}\left(BP_{t}\right)=\frac{BP_{base}-BP_{t}}{0.6\cdot BP_{base}} \end{array}$$ (1)

$$\begin{array}{l} p_{2}\left(BP_{t}\right)=\frac{\left|BP_{base}-BP_{t}\right|}{80} \end{array}$$ (2)

$$\begin{array}{l} p_{3}\left(HR_{t}\right)=\frac{HR_{base}-HR_{t}}{0.3\cdot HR_{base}} \end{array}$$ (3)

$$\begin{array}{l} p_{4}\left(HR_{t}\right)=\frac{HR_{base}-HR_{t}}{40} \end{array}$$ (4)

To compute the overall syncope probability at any given time, we take the maximum of these four relative values (Equation 5). This probabilistic interpretation enables the integration of four independent time series into a single composite series that can be effectively modeled:


$$\begin{array}{l} p\left(t\right)=\max p_{1}\left(BP_{t}\right),p_{2}\left(BP_{t}\right),p_{3}\left(BP_{t}\right),p_{4}\left(BP_{t}\right) \end{array}$$ (5)

To capture the temporal dynamics inherent in the physiological data, we transformed the time-series measurements into a matrix via a sliding window approach. Specifically, we employed a window of 121 consecutive measurements (corresponding to 121 s), where the first 120 measurements serve as input features, and the 121st measurement is the target variable. This configuration is critical for modeling the temporal evolution of the syncope probability signal. This setting was selected empirically based on the following reasoning. Shorter windows produced unstable forecasts, likely because they do not contain sufficient temporal context to capture the relevant hemodynamic trend, whereas substantially larger windows increase the dimensionality and computational burden of the k-nearest neighbors model due to its recurrent forecast implementation and the incorporation of older observations that are less informative for an immediate forecast. Using the above-mentioned window size helped us to balance between the two, leaving the detailed study of the window length impact to our future work.

The predictive modeling involved training the K-NN algorithm, which was selected based on the hypothesis that a patient's future syncope probability can be inferred from the behavior of similar patients. During the hyperparameter optimization, we determined the best model parameters to be *K* = 100 neighbors and the Euclidean distance metric to measure similarity between patient feature vectors. By identifying and leveraging the most similar historical data from a pool of patients—excluding the patient currently under observation—the K-NN model allows us to validate whether syncope events can be predicted based on inter-patient similarity.

Models were trained using data from all patients except the one being observed, employing a leave-one-out cross-validation strategy to ensure that the predictive capability generalizes across different patient profiles. To generate predictions for a ten-minute horizon, forecasts were iteratively fed back through the model, enabling multi-step-ahead prediction and providing a valuable window for potential clinical intervention.

### 2.5 Modeling using an incremental neural network

Our third method is a window-based approach that we have used to try to predict syncope in the first 5 min of the HUTT test, based on the baseline values of HR, ECG, and pressure data. The presented approach directly utilizes the raw data and final HUTT labels, which represent the actual ground truth data.

Given that the provided data was not annotated with syncope occurrences, we first analyzed the provided ECG and pressure data and tried to obtain syncope events from the actual data. We used the definition of positive response to HUTT (see Methods—Head up tilt test). Baseline values are computed as averages in the first 5 min of the HUTT test. Pressure data was already available in the provided data. Heart rate needed to be calculated from ECG data. We have used an open-source library BioSPPy (Hochreiter and Schmidhuber, 1997), which proved to perform the best to detect R-peaks, among other tested open-source libraries. Specifically, the Hamilton segmenter implemented in the BioSPPy (Biosignal Processing toolbox for Python) was employed for automatic R-peak detection from the raw ECG signals sampled at 200 Hz.

Further, we implemented our window-based approach. We used the baseline 5-min data and extracted windows of length 200 (i.e., 1 s of data) with 50% overlap. We labeled the windows according to the final label of the HUTT test. All of the data (pressure, ECG 1, ECG 2) was used, and tensors in the shape of [patient_id, n_windows, window_size, 3] were formed. We have used an LSTM (Long Short-Term Memory)—based model to learn a classification model to classify the windows as being positive or not (Hochreiter and Schmidhuber, 1997). Given the 200 Hz sampling rate, each window of 200 samples corresponds to exactly 1 second of multivariate physiological data.

We used 100 hidden units, a dropout of 50% to prevent overfitting, and a dense, fully connected layer of 100 units, connected to the final dense layer, corresponding to the number of outputs. Categorical cross-entropy was used as a loss, and Adam optimizer for loss optimization. We trained the model for 10 epochs, or as long as the accuracy of the validation set stopped improving.

The model was implemented using the PyTorch deep learning framework (Paszke et al., 2019). Each input sample consisted of a 1-s window of physiological signals sampled at 200 Hz, resulting in tensors of shape (200 × 3), corresponding to 200 time steps and three channels (continuous blood pressure, ECG lead 1, ECG lead 2). The network architecture comprised a single-layer Long Short-Term Memory (LSTM) module with 100 hidden units, followed by a dropout layer (*p* = 0.5) to reduce overfitting. The LSTM output was passed to a fully connected layer with 100 units and ReLU activation, and subsequently to a final fully connected layer with two output neurons. Softmax activation was applied to obtain class probabilities. Model optimization was performed using the Adam optimizer with a learning rate of 1 × 10^−3^. Early stopping was employed by monitoring validation-set classification accuracy; training was terminated when no improvement was observed between consecutive epochs, with a maximum training duration of 10 epochs.

## 3 Results

Head-up tilt test was performed in 105 subjects, who fulfilled the study criteria. Basic demographic and clinical data from the negative (*N* = 53) and positive (*N* = 52) group are shown in Table 1. The Shapiro-Wilk test was used to assess the normality of continuous variable distributions. The *t*-test for equality of means was used for continuous variables and Fisher exact test was used for categorical data. There were no significant differences in those characteristics, including age, body weight, body height, body surface area (BSA), body mass index (BMI), gender, presence of arterial hypertension and hyperlipidemia. Time from the second (“passive”) phase to the onset of syncope (HUTT positive group) was 38 min (33.8–41.3).

**Table 1:** Demographic and clinical characteristics of patients according to the result of head up tilt test (HUTT).

	All participants (*N* = 105)	HUTT poz. (*N* = 52)	HUTT neg. (*N* = 53)	*P* value
Age (years) - median	43 [28, 59]	43 [28.5, 59]	43 [26.5, 58]	0.907
Sex (M/F)	47/58	23/29	24/29	0.914
Body weight (kg)	72.5 (14.6)	73.1 (13.5)	71.9 (15.7)	0.723
Height (cm)—median	170 [164, 177]	171 [165, 177]	167 [162, 177]	0.601
BSA (m2)	1.86 (0.23)	1.87 (0.21)	1.85 (0.26)	0.659
BMI (kg/m2)	24.56 (3.68)	24.64 (4.02)	24.47 (3.37)	0.833
Arterial hypertension (Y/N)	19/86	9/43	10/43	0.836
Hyperlipidemia (Y/N)	13/92	5/47	8/45	0.394

Values of age and height are shown as median [25th percentile, 75th percentile]. Values of body weight, body surface area (BSA) and body mass index (BMI) are shown as mean (SD).

### 3.1 Transformation and processing using FFT

To determine which of our obtained features provide the most predictive performance, we evaluate the three predictive models on four different data set configurations. We first evaluate each model considering only the first 5 minutes of the test, corresponding to only the stabilization phase. We perform a similar test when considering only the first 10 minutes of the passive phase. We then inspect the performance of the models when combining these features together. In our fourth and final experiment, we additionally include the Fourier coefficient differences (Table 2).

**Table 2:** Evaluation of three prediction models on four different data set configurations.

Prediction models	Stabilization phase (5 min)	Passive phase first 10 minutes)	Stabilization and passive phase	Adding coefficient differences
ROC AUC				
Logistic regression	0.64 [0.53, 0.75]	0.64 [0.53, 0.75]	**0.72** [0.62, 0.83]	**0.70** [0.59, 0.81]
Random forest	0.54 [0.43, 0.65]	0.54 [0.43, 0.65]	0.47 [0.36, 0.58]	0.44 [0.32, 0.55]
Gradient boosting	**0.70** [0.60, 0.81]	**0.70** [0.60, 0.81]	0.64 [0.53, 0.75]	0.69 [0.59, 0.79]
Accuracy				
Logistic regression	0.61 [0.51, 0.70]	0.61 [0.51, 0.70]	**0.70** [0.61, 0.78]	**0.68** [0.58, 0.76]
Random forest	0.53 [0.44, 0.63]	0.53 [0.44, 0.63]	0.52 [0.43, 0.62]	0.47 [0.37, 0.56]
Gradient boosting	**0.66** [0.56, 0.74]	**0.66** [0.56, 0.74]	0.57 [0.48, 0.66]	0.60 [0.50, 0.69]

We obtain ROC AUC and accuracy scores using leave-one-out cross-validation for each method. The 95% CI scores are reported in square brackets, and we highlight the best-performing model on each data set with boldface.

Inspecting the statistical differences between the performance of the three methods, we observe that although logistic regression generally outperforms XGBoost, the differences are not statistically significant (all corrected *p*>0.05). Random forest models, on the other hand, performed significantly worse than XGBoost on all four feature sets (all corrected *p* < 0.005), and significantly worse than Logistic Regression when using combined features (both signals: *p* < 0.001; with coefficient differences: *p* < 0.001).

We used implementations of logistic regression and random forests from scikit-learn (v1.6.1) (Fabian, 2011) and use the gradient boosting implementation from the xgboost library (v2.1.4) (Chen and Guestrin, 2016). Confidence intervals and comparisons are computed using the appropriate functions from the confidenceintervals (v1.0.5) and statsmodels (v0.14.4) Python libraries.

### 3.2 Analytical modeling of syncope probability

**Figure 1:**
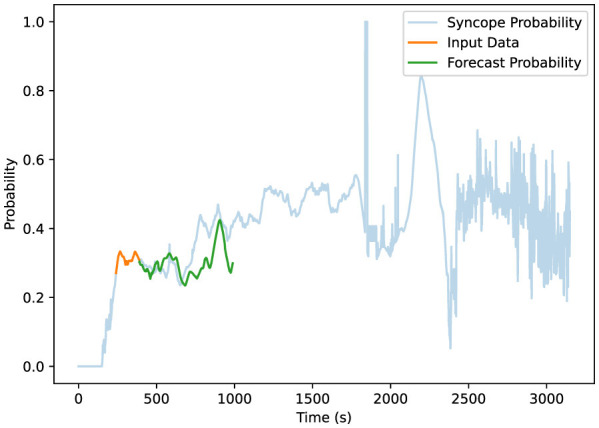
The forecasted probability of syncope (y-axis) over time in seconds (x-axis) for the upcoming ten minutes (0–600 s). The figure shows the true syncope probability (blue curve), the observations used as the input window (orange curve), and the predicted syncope probability based on the input data (green curve).

The results summarized in Table 3 indicate that the model achieves high accuracy for immediate forecasts, with a mean absolute error of 0.06 at t+1 second. The forecasting model was implemented as a k-nearest neighbors regressor using the scikit-learn library, with Euclidean distance as the similarity metric and *k* = 100. Multi-step forecasts were obtained using a recurrent forecasting strategy, in which the most recent prediction was iteratively used as input to generate subsequent predictions until the desired forecast horizon was reached. As the prediction horizon extends to t+30 s and t+60 s, the error increases modestly to 0.09 and 0.11, respectively, suggesting that the model's precision decreases gradually over the short-term forecast interval. At t+120 s, t+180 s, and t+300 s, the error stabilizes at around 0.13, aligning closely with the overall mean absolute error of 0.13, which reflects the model's typical performance. Notably, at t+600 seconds, the error rises to 0.15, highlighting the challenges associated with long-term forecasting. These findings demonstrate a clear trade-off between forecast horizon and prediction accuracy, underscoring the uncertainties in predicting syncope probability over longer time periods. Note, that the mean absolute error (MAE) is reported on the normalized probability scale (dimensionless), as the modeled syncope probability signal is constructed from relative hemodynamic deviations. The example visualization of forecasted syncope probability according to the windowed training data can be seen in Figure 1.

**Table 3:** Mean absolute error according to the prediction horizon.

Forecast time interval	t+1s	t+30s	t+60s	t+120s	t+180s	t+300s	t+600s	Total
Mean absolute error	0.06	0.09	0.11	0.13	0.13	0.12	0.15	0.13

**Figure 2:**
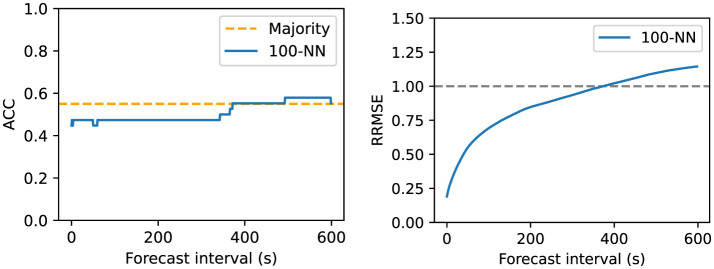
Accuracy and relative root mean squared error (RRMSE) over a 600-second prediction interval.

In addition, we performed further analyses to evaluate classification accuracy and RRMSE (relative root mean squared error) over a 600-second prediction interval (Figure 2). The left graph shows that classification accuracy improves over time, as evidenced by the rising trend above the horizontal orange line that represents random guessing by the majority classifier.

### 3.3 Modeling using an incremental neural network

**Figure 3:**
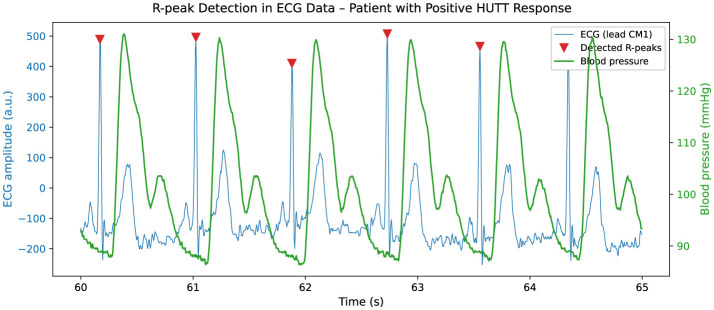
Detection of R-peaks in ECG data using the BioSPPy library (patient with positive response to HUTT). The figure shows a 5-second segment of the ECG signal (blue, left axis) with detected R-peaks (red markers) and the simultaneously recorded continuous blood pressure waveform (green, right axis). The periodic correspondence between ECG R-peaks and the arterial pressure pulse is visible.

**Figure 4:**
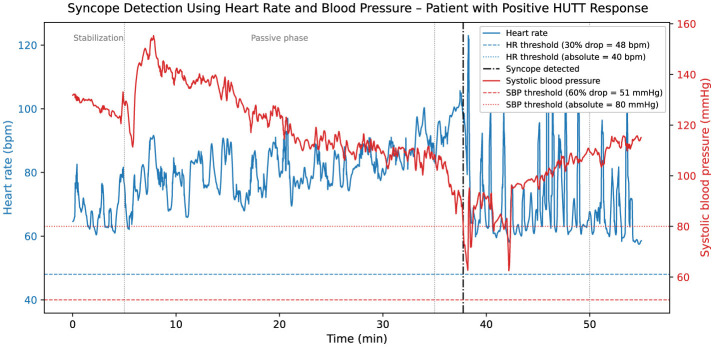
Detection of syncope using heart rate and blood pressure data (patient with positive response to HUTT). Beat-by-beat heart rate (blue, left axis) and systolic blood pressure (red, right axis) are shown across the full 55-min HUTT protocol. Horizontal dashed and dotted lines indicate the syncope detection thresholds (30% HR decrease from baseline and absolute 40 bpm; 60% Systolic Blood Pressure—SBP decrease from baseline and absolute 80 mmHg). The vertical dash-dot line marks the detected onset of syncope during the provocative phase, where a vasodepressor response is evident from the sharp decline in blood pressure.

An example with detected R-peaks is presented in Figure 3. Figure 4 shows an example of syncope detection, given the above-described rules. Note that the used heuristics are not ideal and could result in false detections. The goal was to detect syncope as soon as possible, and the medical expert also suggested that we focus on the first 5 minutes of data instead. We thus only used the global HUTT-level based labels, which are reliable and were experimentally proven.

We divided the training set into training, validation, and test sets independently across patients. 10 positive and 10 negative patients were left out for both the validation and test set. Performance on the validation set was evaluated directly on the extracted windows and the results were not aggregated to the patient level. We performed training 10 times to get an average result. For the evaluation of the test set, we needed to aggregate the window-based results. We used different strategies (e.g., mean, max) to aggregate the classification results in terms of the probability of all the windows. Overall results on the validation set achieved an accuracy 67% of when using the mean pooling, while on the test set, the performance dropped to 62%. All the results are reported as the average over 10 runs.

## 4 Discussion

In this study, we explored three distinct approaches to predict the outcome of the head-up tilt test (HUTT) in patients with recurrent syncope. Among the evaluated models, the analytical modeling approach—using a k-nearest neighbor regression to forecast syncope probability from transformed physiological signals–demonstrated the most consistent short-term forecasting performance, with mean absolute errors remaining below 0.13 for predictions up to 300 s in advance. Additionally, gradient boosting models applied to frequency-domain features also showed promising classification performance, achieving ROC AUC scores of up to 0.70. These findings highlight the potential of early physiological signal analysis for predicting vasovagal syncope.

**Transformation and processing using FFT:** The evaluation results (listed in Table 2 show that both the logistic regression and gradient boosting models perform well, achieving an average AUC score of around 0.7, while the random forest model performs significantly worse. While it appears that the gradient boosting models are able to achieve good predictive accuracy based on the Fourier coefficients of the stabilization phase alone, the logistic regression model appears to benefit from the inclusion of both phases. Including the coefficient differences appears to yield little additional information, however, several of these were assigned a high feature importance by both the logistic regression and gradient boosting methods. This may indicate a high degree of redundancy between the features. It is, however, encouraging that gradient boosting models achieve good performance based on the stabilization phase alone, as this could lead to the development of early warning systems, flagging potentially at-risk patients.

The present approach differs from the results of other investigators in several important ways. While Hussain et al. (2021) developed a classification system that achieves nearly 100% accuracy, it is important to note that, in their work, they do not attempt to forecast the future occurrence of syncope events, but rather, given an observational data of a HUTT test administration, determine whether a syncope event occurred or not. Our approach is conceptually similar to our prior work (Klemenc and Štrumbelj, 2015) in which we found the performance of linear models similar to a naive majority classifier baseline. Unlike in our prior work, in which we used a small number of derived predictors, we here apply these models directly to high-magnitude FFT coefficients. We think that the increased performance in our present work is likely due to information loss when summarizing the time-series data in compressed feature descriptors, while we here allow models to learn from the full spectrum of the signals.

Our FFT-based approach converts physiological signals into the frequency domain and uses machine learning classifiers (logistic regression, random forest, gradient boosting) to foresee HUTT outcomes. This approach is conceptually similar to the study by Lee et al. (2024), who studied 26 machine learning models, including XGBoost, to predict vasovagal syncope by heart rate variability (HRV) features. Their best-performing model (XGB classifier) reached an AUC of 0.801 and could predict syncope events approximately three minutes prior to onset. Although their approach did not depend on full-spectrum frequency transformation, the effort on early predictive modeling using time-series features and tree-based classifiers corresponds closely to our approach.

A related study by Ferdowsi et al. (2024) utilized physiological data from tilt testing and reported excellent performance (ROC AUC = 0.954) using a combination of KNN imputation and support vector machine (SVM) classification. While this method also depends on extracted features from hemodynamic signals, it did not use raw or frequency-domain data directly. In contrast, our method leverages FFT-derived coefficients as input features, potentially maintaining a richer representation of the signal dynamics.

**Analytical Modeling of Syncope Probability:** The results of analytical modeling indicate that the binary decision of whether a patient will experience syncope becomes increasingly reliable as time progresses. This is likely because most syncope events occur relatively early, making the prediction problem simpler over time. However, this measure only reflects the accuracy of the binary decision and does not capture the magnitude of errors in predicting syncope probability. In contrast, the increasing RRMSE highlights the growing difficulty of long-term forecasting. Our results suggest that predictions remain meaningful up to approximately 300 -400 seconds, beyond which the reliability of the forecasts diminishes significantly.

Our analytical framework models the evolution of syncope probability using normalized physiological features derived from hemodynamic parameters and predicts future values using a KNN regressor. This concept of continuous risk modeling finds parallel in the study by Meyer et al. (2011), a similar concept of continuous risk modeling was tested. They proposed a syncope prediction alarm based on heart rate (HR) and pulse arrival time (PAT). The developed algorithm successfully predicted syncope events in all positive cases, with an average time between the prediction alarm and the occurrence of syncope of 99 ±108 seconds. Although their model depends on slope detection and rule-based thresholds, it supports the feasibility of early warning systems based on evolving physiological signals. Because the kNN-based forecasting model relies on inter-patient similarity, subgroup-restricted modeling (e.g., sex-specific models) could theoretically improve physiological homogeneity. However, given the limited cohort size and the use of leave-one-out cross-validation, restricting the neighbor pool would substantially reduce the number of available reference trajectories per fold, potentially increasing model variance and instability. In our cohort, no significant sex differences were observed between positive and negative HUTT groups, and epidemiological studies report comparable syncope incidence between men and women. Therefore, we adopted a population-level modeling strategy to preserve statistical robustness. Future studies with larger datasets should investigate whether subgroup-specific similarity modeling provides incremental benefit.

Another relevant contribution is the study by Mereu et al. (2013), which evaluated the time derivative of the RR interval and systolic blood pressure and found it capable of predicting syncope approximately 44 seconds in advance, with sensitivity and specificity exceeding 86%. Their derivation of predictive signals from relative changes reflects our transformation of blood pressure and heart rate measurements into the proposed probability function.

In a broader sense, the probabilistic modeling approach we employ also shares conceptual similarity with the work of Ho et al. (2016), who found that early changes in blood pressure during HUTT were significantly associated with final outcomes. They developed an algorithm based on age and blood pressure with an overall accuracy of 79.8%. While their model was deterministic and based on statistical interaction terms, it demonstrates the predictive utility of early physiological changes–an assumption that also underlies our modeling framework. In contrast to their work, our approach constructs a continuous, time-dependent probability function derived directly from ESC-defined hemodynamic criteria (Brignole et al., 2018). Rather than performing static early classification, we model the temporal evolution of syncope probability using a sliding-window KNN regression framework and perform multi-step forecasting up to 600 seconds into the future. This design enables dynamic risk monitoring and gradual risk escalation assessment, representing a fundamentally different modeling philosophy from binary classification approaches.

**Modeling using Incremental Neural Network:** The results of the incremental neural network approach indicate moderate predictive performance when applied to early-stage HUTT data. While the model demonstrates reasonable classification accuracy on the validation set, its generalization to the test set shows a noticeable decline, highlighting the challenges of inter-patient variability and the limited discriminative power of early-phase physiological signals alone. The use of short, overlapping time windows provides temporal granularity, but may also introduce redundancy and reduce model robustness. Note that only the first 5 minutes of the HUTT test was used to train and evaluate the model, which represents the most challenging scenario and at the same time, would present a significant value in terms of the reduction of risk of performing the test on a patient. Moreover, reliance on heuristic labeling based on predefined thresholds for syncope detection could lead to label noise, impacting learning efficacy, but could increase the training set and offer greater discriminative power for the model. We opted not to use the specific labeled regions and instead used HUTT-level labels to reduce the risk of the model simply learning the underlying labeling heuristics. Despite the mentioned limitations, the approach underscores the potential of neural architectures for processing raw physiological signals and motivates further refinement through improved annotation strategies and data augmentation techniques.

Myrovali et al. (2021) developed a multilayer perceptron neural network (MPNN) to classify syncope based on HRV features and systolic blood pressure recorded at rest. Their best model achieved an accuracy of 89.7%, indicating the utility of neural architectures for detecting latent patterns in early physiological signals. However, while their features were derived from wavelet bispectrum analysis and used a limited patient cohort, our method processes the raw signal directly and models the temporal structure of the data through recurrent connections.

**Limitations of the study:** Although we implemented three distinct modeling approaches, their performance was evaluated using different metrics, such as ROC AUC, mean absolute error, and classification accuracy. This heterogeneity complicates direct comparison across methods and may obscure subtle performance trade-offs. Additionally, each approach comes with inherent constraints: the FFT-based models may include redundant or noisy features and offer limited interpretability; the analytical model assumes inter-patient similarity, which may not generalize well to more heterogeneous populations; and the neural network model relies on global HUTT-level labels, introducing potential label noise and limiting temporal precision. These challenges highlight the need for unified evaluation frameworks, refined annotation strategies, and further validation to support broader clinical applicability.

## Data Availability

The ECG and arterial pressure datasets used in this study are publicly available: https://www.sbng.si/file/2215652716491232_tilt-n-raziskava-anonimno2025-10-20.zip.
